# Dye-decolorizing peroxidases in *Irpex lacteus* combining the catalytic properties of heme peroxidases and laccase play important roles in ligninolytic system

**DOI:** 10.1186/s13068-018-1303-9

**Published:** 2018-11-08

**Authors:** Xing Qin, Huiying Luo, Xiaoyu Zhang, Bin Yao, Fuying Ma, Xiaoyun Su

**Affiliations:** 1 Department of Biotechnology, College of Life Science and Technology, Huazhong University of Science and Technology, Wuhan, 430074 China; 2 Key Laboratory for Feed Biotechnology of the Ministry of Agriculture, Feed Research Institute, Chinese Academy of Agricultural Sciences, No. 12 South Zhongguancun Street, Beijing, 100081 China

**Keywords:** Dye-decolorizing peroxidase, White rot fungi, *Irpex lacteus*, Lignin biodegradation, Mediator

## Abstract

**Background:**

The white rot fungus *Irpex lacteus* exhibits a great potential in biopretreatment of lignocellulose as well as in biodegradation of xenobiotic compounds by extracellular ligninolytic enzymes. Among these enzymes, the possible involvement of dye-decolorizing peroxidase (DyP) in lignin degradation is not clear yet.

**Results:**

Based on the extracellular enzyme activities and secretome analysis, *I. lacteus* CD2 produced DyPs as the main ligninolytic enzymes when grown in Kirk’s medium supplemented with lignin. Further transcriptome analysis revealed that induced transcription of genes encoding DyPs was accompanied by the increased expression of transcripts for H_2_O_2_-generating enzymes such as alcohol oxidase, pyranose 2-oxidase, and glyoxal oxidases. Meanwhile, accumulation of transcripts for glycoside hydrolase and protease was observed, in agreement with abundant proteins. Moreover, the biochemical analysis of *Il*DyP2 and *Il*DyP1 confirmed that DyPs were able to catalyze the oxidation of typical peroxidases substrates ABTS, phenolic lignin compounds DMP, and guaiacol as well as non-phenolic lignin compound, veratryl alcohol. More importantly, *Il*DyP1 enhanced catalytic activity for veratryl alcohol oxidation in the presence of mediator 1-hydroxybenzotriazole, which was similar to the laccase/1-hydroxybenzotriazole system.

**Conclusions:**

The results proved for the first time that DyPs depolymerized lignin individually, combining catalytic features of different peroxidases on the functional level. Therefore, DyPs may be considered an important part of ligninolytic system in wood-decaying fungi.

**Electronic supplementary material:**

The online version of this article (10.1186/s13068-018-1303-9) contains supplementary material, which is available to authorized users.

## Background

Lignin is the second most abundant constituent of lignocellulosic biomass, amounting to 15–30% by weight or up to 40% by energy [1]. The degradation of lignin represents a key step for carbon recycling in the land ecosystems, as well as a critical issue for cost-effective lignocellulosic biofuels and bio-based chemicals [2]. However, due to the complex and random phenylpropanoic polymeric structure, lignin is highly recalcitrant toward chemical and biological degradations [3], resulting in lignocellulosic waste and environment pollution. White rot fungi, a large group of wood-decaying basidiomycetes, are able to completely decompose lignin into carbon dioxide and water by extracellular ligninolytic enzymes, which include an array of heme peroxidases and oxidases [4]. Among them, the heme peroxidases, such as manganese peroxidase (MnP), versatile peroxidase (VP), lignin peroxidase (LiP), and laccase (Lac) have been considered to play important roles in lignin degradation [5–7].

Dye-decolorizing peroxidase (DyP) is a member of the novel heme peroxidase family (DyP-type peroxidase superfamily), showing no homology to classic fungal heme peroxidases including MnP, VP, and LiP [8, 9]. So far only eleven fungal DyPs have been purified and characterized [10]. Compared with classic fungal heme peroxidases, the specific feature of all DyP is the ability to oxidize synthetic high redox potential dyes of the anthraquinone type [8]. DyP can oxidize phenolic compounds, such as 2,6-dimethoxyphenol and guaiacol [7]. Recently, there are a few reports about its catalytic ability to non-phenolic lignin model compound veratryl alcohol (VA) and Mn^2+^, which is attributed to high redox potential peroxidase LiP/VP and MnP, respectively [7, 10, 11]. These findings indicate that DyP might be an important part of ligninolytic system in white rot fungi, although biological roles of DyP are ambiguous in terms of different substrate specificities.

*Irpex lacteus* is a white rot fungus with a significant potential for various biotechnological applications such as bioremediation of organopollutants in water and soil environments and biopretreatment of lignocellulose [12, 13]. Its biotechnological applications were attributed to the extracellular ligninolytic enzymes, including MnP, LiP, laccase-like, and DyP [14–18]. Our preliminary work demonstrated *I. lacteus* CD2 could degrade all kinds of lignocellulose and dyes [13–15]. Genome analysis reveals that *I. lacteus* CD2 has seven *mnp* genes, two *lip* genes, and four *dyp* genes, without *lac* gene [14]. Compared with MnP, the main ligninolytic enzyme of *I. lacteus*, DyP is scarcely known for catalytic properties and substrate specificities, especially in lignin degradation. Herein, the main ligninolytic enzymes of *I. lacteus* CD2 grown in lignin medium were determined, combining extracellular enzyme activities and secretome analysis. Furthermore, the mechanisms of lignin degradation by the main ligninolytic enzymes DyPs were elucidated using transcriptomics and biochemical analysis.

## Results and discussion

### Major extracellular proteins and ligninolytic enzymes of *I. lacteus* in lignin medium

**Fig. 1:**
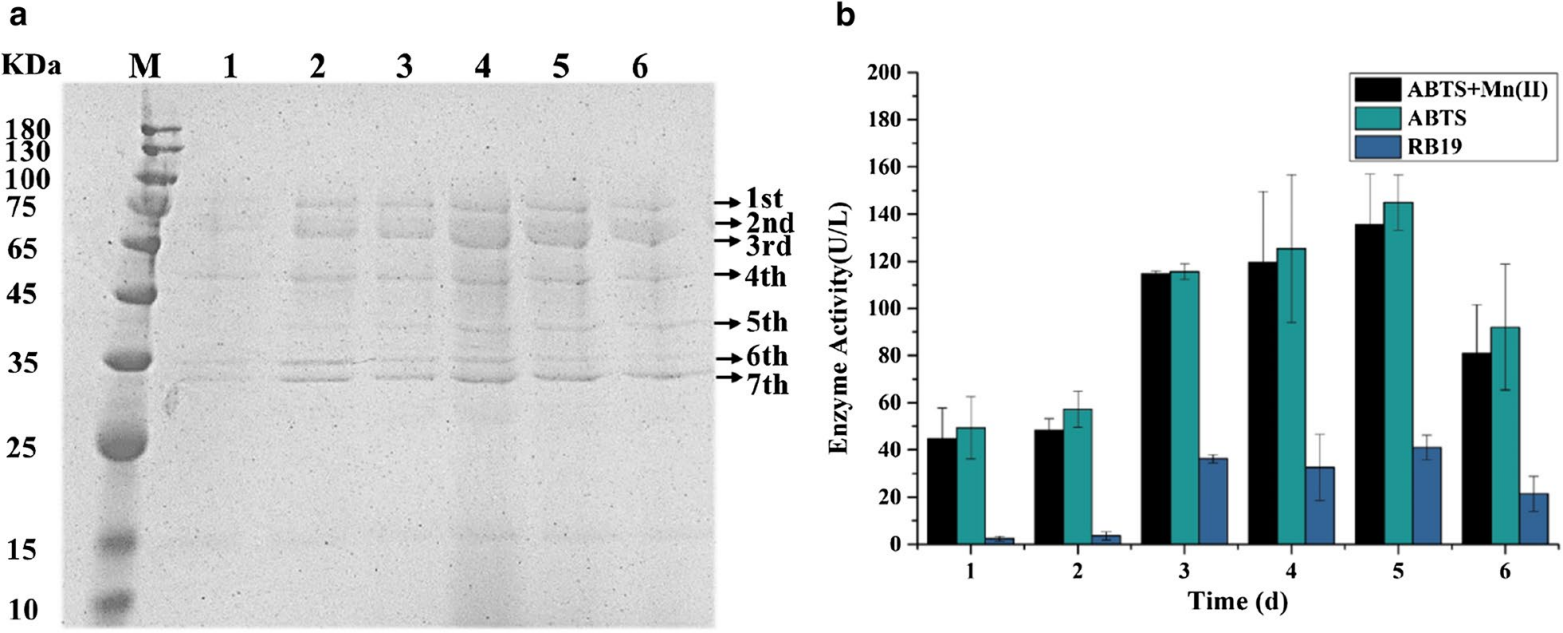
Time course analysis of crude protein by SDS-PAGE (**a**) and peroxidases activities (**b**) in *I. lacteus* CD2 grown in Kirk’s medium supplemented with lignin. Lanes: M, the protein molecular mass marker; 1–6, concentrated culture supernatant from the 1st to the 6th day. ABTS + Mn(II), ABTS, and RB19 represented total ligninolytic enzyme activities, manganese-independent peroxidase activity, and DyP activity, respectively

The SDS-PAGE analysis showed there were seven main bands within 30–75 kDa in extracellular proteins of *I. lacteus* in lignin medium (Fig. 1a). At the same time, extracellular ligninolytic enzyme activities were detected (Fig. 1b). There was no significant difference between total ligninolytic enzymes activity and manganese-independent peroxidases (MiPs) activity, which suggested MiPs were the main extracellular ligninolytic enzymes of *I. lacteus* CD2 in lignin medium, and MnP activity might be weak or negligible. DyPs are one of MiPs and showed the similar trend to that of MiPs in *I. lacteus* CD2 grown in lignin medium (Fig. 1a), suggesting that DyPs might be the main extracellular ligninolytic enzymes of *I. lacteus* CD2 in lignin medium. MiPs including DyP were rapidly induced since lignin and lignin-derived aromatic compounds were the most efficient inducers of ligninolytic enzymes [19]. In this study, MiPs activities increased rapidly on day 3 and obtained peaks on day 5, with maximal activities of 144.9 U/L and 41.1 U/L against ABTS and RB19, respectively (Fig. 1a). The change of protein contents was consistent with extracellular ligninolytic enzymes activities over time.

In order to determine the corresponding MiPs produced in lignin degradation, the seven gel bands on day 3 in SDS-PAGE were recovered and subjected to nano LC/MS–MS analysis. The results confirmed that DyPs were the main extracellular ligninolytic enzymes of *I. lacteus* in lignin medium (Table 1, Additional file 1). In parallel, *I. lacteus* secreted different oxidoreductases including aryl alcohol oxidase (AAO), copper radical oxidase, and cellobiose dehydrogenase (CDH) (Table 1), which can generate H_2_O_2_ required for the action of extracellular peroxidases [20].

**Table 1:** Major extracellular proteins identified from the SDS-PAGE bands of culture supernatant of *I. lacteus* CD2 in lignin-Kirk’s medium

Number	Accession	− 10lgP	Coverage (%)	Peptides	Unique	PTM	Avg. Mass	Description
1st	0809.852	377.71	57	34	34	Y	80,666	Glutaminase
	0810.199	278.1	28	15	15	N	81,041	Glucan 1,3-beta-glucosidase
2nd	0806.17	329.07	27	13	13	Y	63,771	Aryl-alcohol oxidase
	0821.105	243.71	20	6	5	N	52,706	Dye-decolorizing peroxidase
3rd	0821.105	388.5	28	10	8	N	52,706	Dye-decolorizing peroxidase
	0807.1163	240.17	18	7	7	Y	47,202	Alpha-galactosidase
4th	0811.256	326.61	32	10	10	Y	51,332	Alpha-amylase
	0816.66	315.51	34	14	13	Y	51,687	Oxalate decarboxylase
5th	1079.46	363.17	18	6	5	N	42,231	Endo-1,6-alpha-mannosidase
	0807.283	265.31	12	7	6	N	42,718	Polyporopepsin
6th	0816.57	277.37	27	9	9	N	39,674	Endopolygalacturonase
	0807.283	271.85	12	7	6	N	42,718	Polyporopepsin
7th	0807.283	580.05	53	17	16	N	42,718	Polyporopepsin
	0925.239	446.02	36	11	10	N	43,170	Polyporopepsin

In addition to ligninolytic enzymes, some proteins involving in fungal growth, such as glycosidase hydrolases (including chitinases, glucanases, mannosidases and so on) and proteases were identified in the extracellular proteins. Chitinases, glucanases, and mannosidases are involved in hyphal cell wall biosynthesis [21]. Proteases such as polyporopepsin, aspartic protease, and glutaminase are implicated in protein degradation and supplying nitrogen for fungal growth [22, 23]. Glycoside hydrolases are essential for cell wall synthesis and cell wall integrity, involving in protein maturation and transport, such as N-linked glycoproteins processing or carbohydrate structural degradation [24].

### Comparative transcriptome analysis of *I. lacteus* grown in lignin and glucose medium

**Fig. 2:**
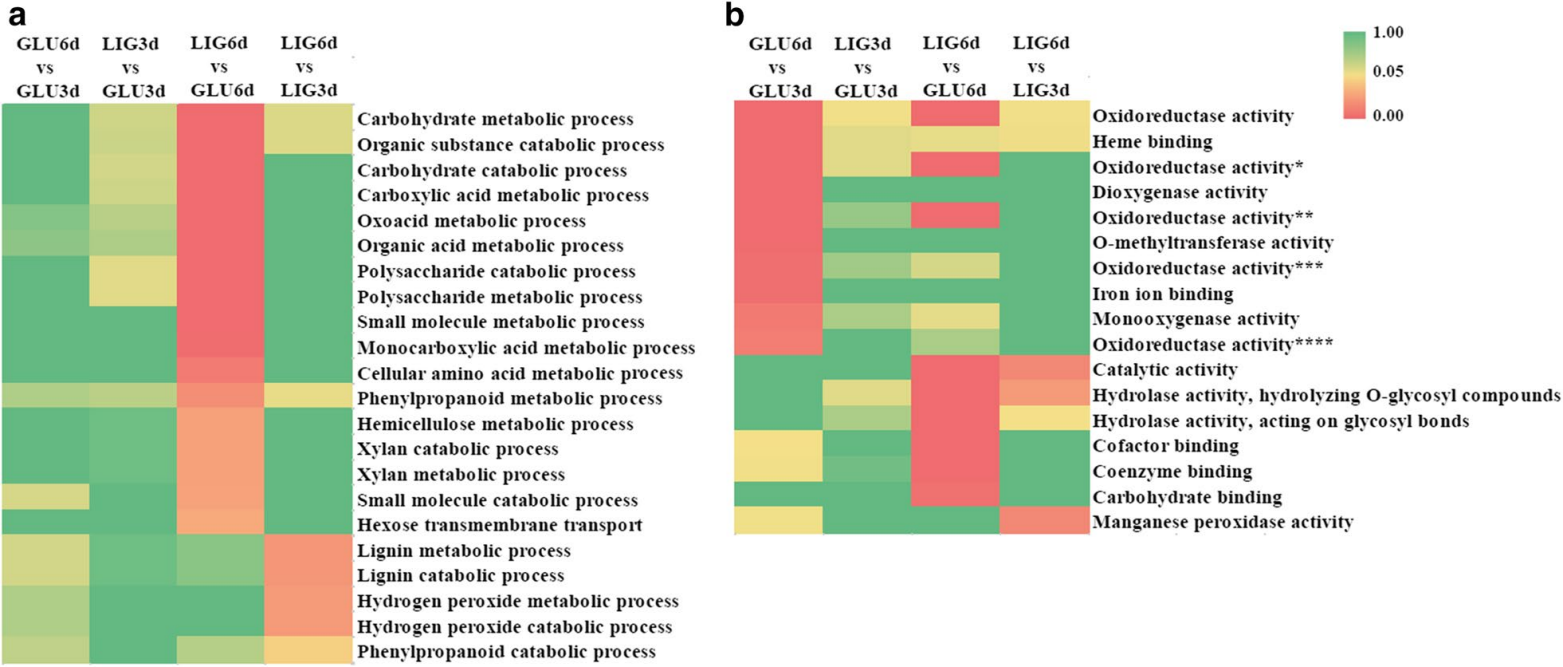
GO-enrichment analysis of differently expressed genes in four pairwise comparisons: GLU6d versus GLU3d, LIG3d versus GLU3d, LIG6d versus GLU6d, and LIG6d versus LIG3d. Biological process (**a**) and molecular function (**b**) involved in lignocellulose-degrading. GLU3d/GLU6d represented 3 days/6 days of cultivation in glucose medium, LIG3d/LIG6d represented 3 days/6 days of cultivation in lignin medium

A total of 10,167 genes were determined from the transcriptomes of four samples: LIG3d (3 days in lignin), GLU3d (3 days in glucose), LIG6d (6 days in lignin) and GLU6d (6 days in glucose). To identify key genes and pathways associated with lignin degradation, four pairwise comparisons were performed, including LIG3d versus GLU3d, LIG6d versus GLU6d, LIG6d versus LIG3d , GLU6d versus GLU3d. The results indicated that 4603 and 3816 genes showed at least twofold differences in comparisons of groups LIG3d versus GLU3d, and LIG6d versus GLU6d, respectively, whereas only 300 genes exhibited not less than twofold differences in the comparison of group LIG6d versus LIG3d. Moreover, GO-enrichment analysis between LIG6d versus GLU6d and LIG6d versus LIG3d revealed that these differential expression genes were significantly enriched in lignocellulose-degrading process, including lignin, carbohydrate, polysaccharide, hemicellulose, hydrogen peroxide, and phenylpropanoid metabolic/catabolic process (Fig. 2a). Besides, genes encoding oxidoreductase, heme binding, peroxidase, monooxygenase, and glycoside hydrolase (GH) were also significantly enriched (Fig. 2b). These results were in agreement with that of protein analysis. Since white rot fungi were able to produce different heme peroxidases in synergy with oxidases, and each one might contribute in different ways to the final degradation of lignin [25]. The synergetic effects of different enzymes play vital roles in depolymerizing lignin by *I. lacteus* CD2.

### Ligninolytic enzyme system

**Fig. 3:**
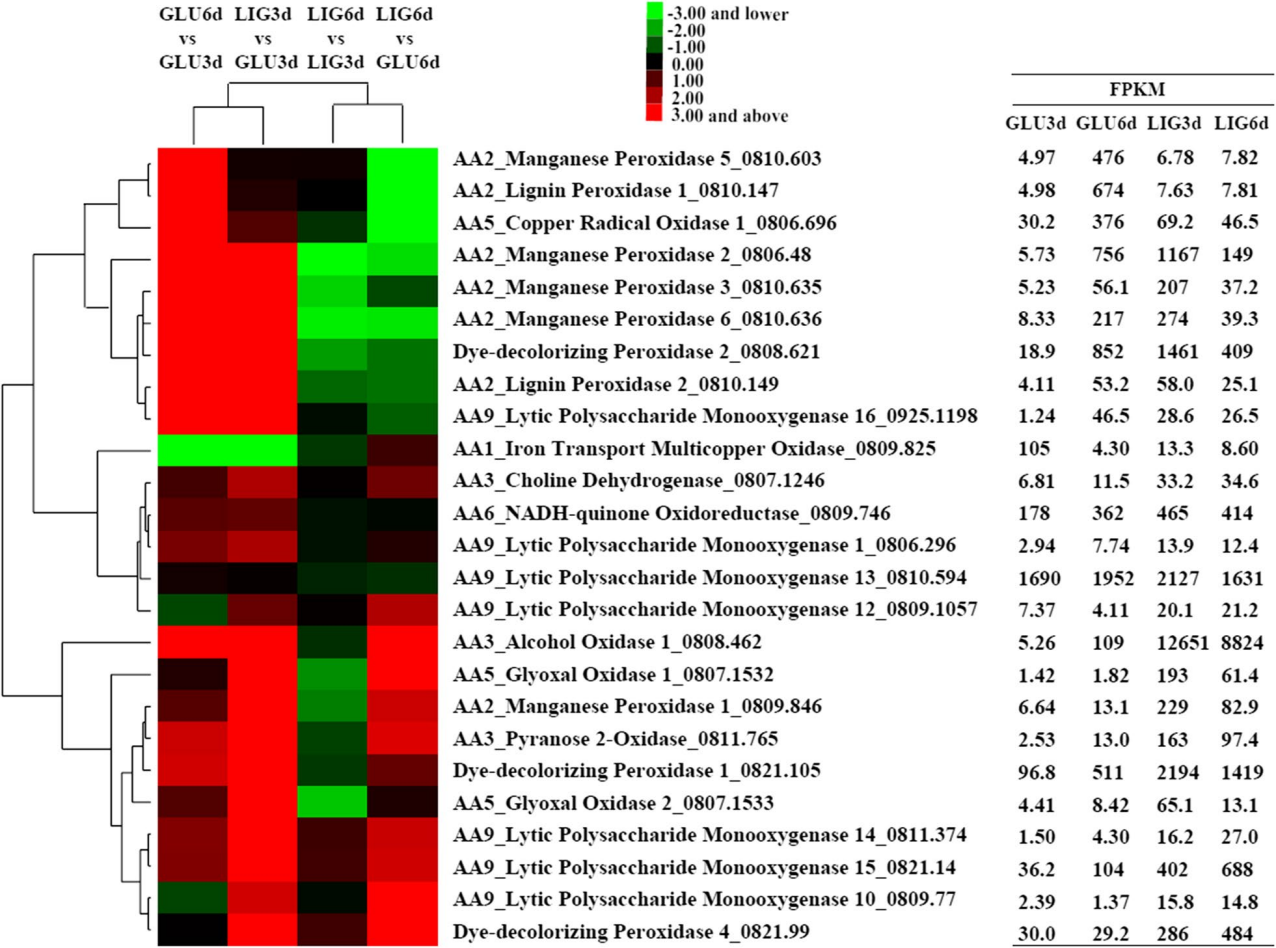
Differential expression analysis of lignin-degrading enzyme-encoding genes in comparisons of GLU6d versus GLU3d, LIG3d versus GLU3d, LIG6d versus LIG3d, and LIG6d versus GLU6d. GLU3d/GLU6d represented 3 days/6 days of cultivation in glucose medium; LIG3d/LIG6d represented 3 days/6 days of cultivation in lignin medium

Various lignin-degrading peroxidases were significantly differentially expressed along with H_2_O_2_-generation enzymes in *I. lacteus* in lignin medium (Fig. 3). The transcripts encoding DyPs were highly expressed in LIG3d and LIG6d, which were in agreement with extracellular peroxidases activities and protein profiles. Compared with in GLU3d, *Il*DyP1 and *Il*DyP2 in LIG3d were induced by 22.7- and 77.3-fold, then decreased by 1.5- and 3.6-fold in LIG6d, respectively. *Il*DyP4 gene was upregulated by 9.5-fold and further increased by 1.7-fold in LIG6d. Also, one LiP and four MnP transcripts were abundant in LIG3d, especially for *Il*MnP2. Notably, although the respective activities were not detected in the extracellular culture filtrates, MnP and LiP isoenzymes were still expressed to different extents in different stages. The similar cases were also reported in *Pleurotus ostreatus* that MnP and Lac with high transcription levels were not found in extracellular proteins, which might be inefficient secretion and the action of specific proteases [26, 27]. In addition, transcripts for one alcohol oxidases (AOX) gene, one pyranose-2-oxidase (POX) gene, and two glyoxal oxidase (GLOX) genes were significantly accumulated in LIG3d. AOX gene was upregulated by 2405.1-fold in LIG3d and decreased by 1.4-fold in LIG6d. The membrane-anchored AOX, proposed to be an extracellular source of H_2_O_2_ during lignin degradation [20, 28], was the most abundant transcript in H_2_O_2_-generation enzymes. POX gene transcript also accumulated in LIG3d and LIG6d. POX was not found in extracellular proteins in some studies, which was attributed to its location in the periplasmic space and associated with membranous materials [29, 30]. Besides, *Il*GLOX1 and *Il*GLOX2 were also induced by 135.9- and 14.8-fold in LIG3d, respectively. These suggested that AOX, POX and GLOX were implicated in providing H_2_O_2_ required for DyP activity.

### Carbohydrate metabolism, nitrogen metabolism and related enzymes

In addition to oxidoreductases, genes encoding carbohydrate active enzymes (CAZYs) as well as other proteins were upregulated in lignin medium. 47 CAZYs genes of glycoside hydrolase, carbohydrate esterase (CE) or polysaccharide lyase (PL) families had significantly different transcript abundance in LIG3d relative to GLU3d (Additional file 2). The GHs mainly consisted of cellulose-degrading enzymes (GH6), hemicellulose-degrading enzymes (GH10/GH43/GH51), and pectin-degrading enzymes (GH28/GH78/GH88). These enzymes might be to hydrolyze a small amount of carbohydrate remained in lignin, and required for fungal growth. In particular, genes related to CAZYs including trehalase, chitinase, and mannosidase were upregulated. These enzymes might liberate carbon from major storage compound trehalose and be involved in fungal hyphal cell wall biosynthesis for growth [21, 31].

Numerous genes involved in mobilizing and recycling nitrogen were also expressed, including oligopeptide transporter, nucleoside transporter, acetamidase, amino acid permease, amine oxidase, arginase, aspartokinase, methionine synthase, nitrilase, and proteases (Additional file 3). In accordance with the abundance in the extracellular culture filtrates, polyporopepsins, and aspartic protease genes were early induced by lignin (LIG3d). Notably, polyporopepsin gene 0925.239 had the most expression level among extracellular proteases. In addition, acetamidases and amino acid permease genes were significantly differentially accumulated in lignin medium (LIG3d and LIG6d).

The above results suggested that DyPs were the main extracellular ligninolytic enzymes of *I. lacteus* in lignin medium, while in lignocellulose medium MnPs played key roles [14]. Therefore, DyPs were purified from lignin medium for studying their characterization and catalytic properties.

### Purification and characterization of DyPs from *I. lacteus* in lignin medium

**Fig. 4:**
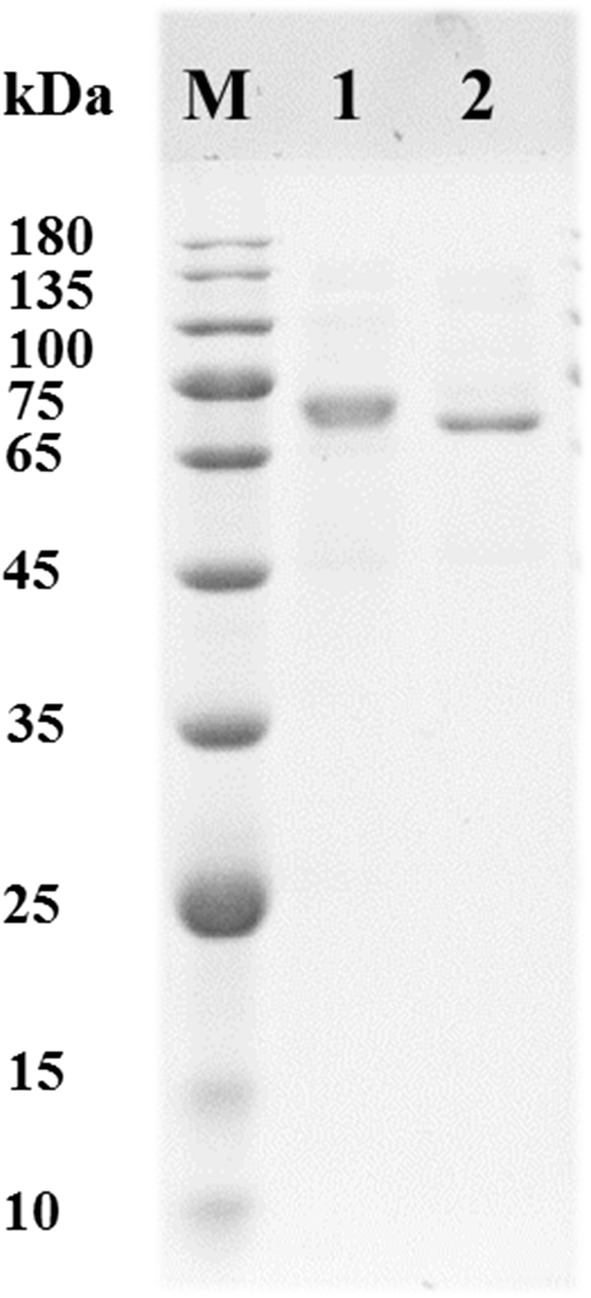
Purified *Il*DyP2 and *Il*DyP1 were analyzed by SDS-PAGE. Lanes: M, the protein molecular mass marker; 1, the purified *Il*DyP2; 2, the purified *Il*DyP1

Two DyP isoenzymes were purified to homogeneity from the liquid cultures of *I. lacteus* grown in alkali lignin medium for 3 days (Fig. 4). The molecular mass of *Il*DyP2 and *Il*DyP1 were about 74 kDa and 72 kDa, respectively. Like other classic heme peroxidases (e.g., MnP, VP, and LiP), DyPs also contain heme group judging from the absorbance at 410 nm [5, 16, 32]. The Rz (A_410_/A_280_) ratios of purified DyPs were 2.5 and 1.0, respectively. Meanwhile, in order to identify corresponding DyP isoenzymes expressed in *I. lacteus* CD2, purified DyPs were separated from SDS-PAGE to digested for peptide mass fingerprinting analysis. Based on the number of unique peptides matched in DyPs from *I. lacteus*, *Il*DyP2 and *Il*DyP1 corresponded to genes 0808.621 and 0821.105, respectively (Table 2).

**Table 2:** Matching peptides with DyPs from *I. lacteus* after *Il*DyP2 and *Il*DyP1 trypsin digestion and PMF analysis

Protein accession	Peptide	Unique	Mass	*m/z*	Start	End
*Il*DyP2_0808.621	R.TNDPVTNRPSWALDGSFLVFR.K	Y	2391.2	798.07	254	274
	K.SEPLGLDPVIGQGTR.Q	Y	1537.81	513.61	443	457
	R.GLAFVEYQSVISNGFR.F	Y	1785.9	893.96	411	426
	R.NNDFNYIHPGEDLTTDETR.C	Y	2249.98	1126	340	358
	K.LKQLVPEFHK.W	Y	1237.72	619.87	276	285
	K.QLVPEFHK.W	Y	996.54	499.28	278	285
	R.C(+ 57.02)*PFTAHVR.K	Y	986.48	494.25	359	366
	R.QTFGLDPR.N	Y	932.47	467.24	458	465
	R.KLKQLVPEFHK.W	Y	1365.81	456.28	275	285
	K.GTNVDGVFLIGSDDVTTTNQYRDDLK.A	Y	2842.36	948.46	167	192
	R.WNSGAPIDLTPDVDDPTLGNDPQR.N	Y	2592.21	1297.11	316	339
*Il*DyP1_0821.105	R.TQQEVWANAAAFPFGK.T	Y	1763.86	882.94	424	439
	R.AGTPYGPELTDDESSSGTTSTDR.G	Y	2343	1172.51	385	407
	K.SGAPIDLSPDVDDASLGTDPQR.N	Y	2225.04	1113.53	317	338
	K.TESIGLDPVIGQGDR.T	Y	1555.78	778.9	440	454
	R.DLESVIGHANHAIR.A	Y	1530.79	766.4	371	384
	K.QLVPEFNK.W	Y	973.52	487.77	277	284

**Fig. 5:**
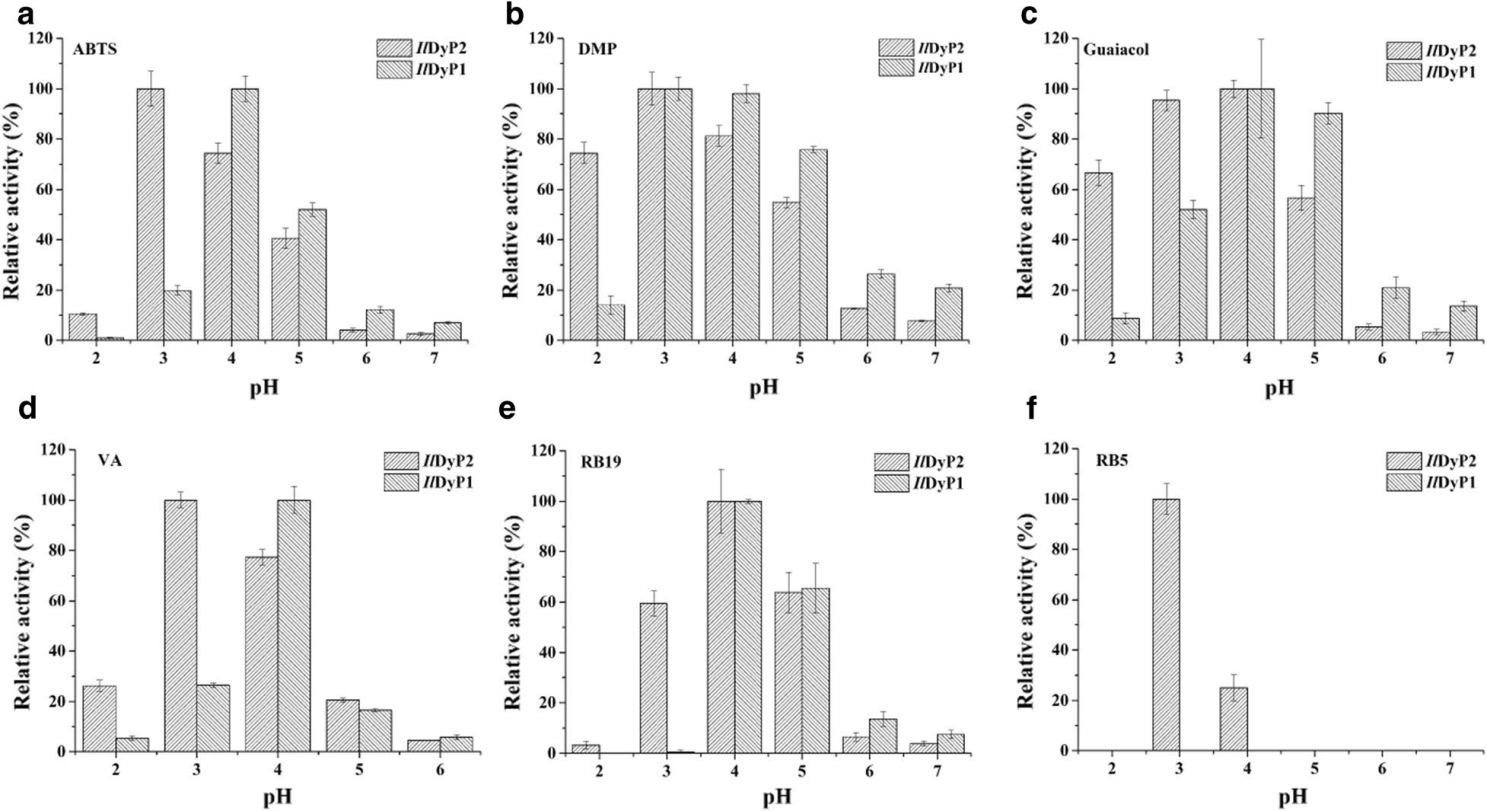
Optimum pH of the purified IlDyP2 and IlDyP1 oxidizing different substrates: ABTS (**a**); lignin model compounds DMP (**b**), guaiacol (**c**) and VA (**d**); high redox potential dyes RB19 (**e**) and RB5 (**f**)

The optimal pH for *Il*DyP2 and *Il*DyP1 was determined in pHs ranging from 2 to 7 among six different substrates, including high redox potential dyes (RB19 and RB5), lignin model compounds (DMP, guaiacol, and VA) and ABTS (Fig. 5). *Il*DyP2 and *Il*DyP1 showed the same optimal pH 4.0 for oxidation of RB19 and guaiacol, in agreement with DyP for RB19 from *I. lacteus* CCBAS 238 [16]. *Il*DyP2 showed acidic optima for guaiacol (67%, 95%, and 57% of residual activities at pH 2, pH3, and pH 5, respectively), while *Il*DyP1 was found to show less acidic optima (9%, 52%, and 90% of residual activities at pH 2, pH3, and pH 5, respectively). However, only *Il*DyP2 could oxidize RB5 in the narrow pH range from 3.0 to 4.0, which was slightly different from DyP in *I. lacteus* CCBAS 238 with optimal pH 3 and retaining 60% of residual activity at pH 2 for RB5. The optimal pH of 3.0 and 4.0 for *Il*DyP2 and *Il*DyP1 against VA and ABTS, respectively, and pH 4 for *Il*DyP1 could retain 80% of residual activity against VA and ABTS, while DyP in *I. lacteus* CCBAS 238 had optimal pH 2 for VA and pH 3 for ABTS, and retained 10% and 60% of residual activities at pH 4 for VA and ABTS, respectively. *Il*DyP2 and *Il*DyP1 showed the same optimal pH 3.0 for DMP, in accordance with DyP in *I. lacteus* CCBAS 238 [16]. *Il*DyP2 showed acidic optima of DMP (74%, 81%, and 55% of residual activity at pH 2, pH4, and pH 5, respectively), while *Il*DyP1 was found to show less acidic optima (14%, 98% and 76% of residual activity at pH 2, pH 4, and pH 5, respectively). The optimal values of DyPs in this study were very near to those of DyP from *Auricularia auricula*-*judae* (pH 3.5 for RB19, pH 2.5 for VA, and pH 3.0 for DMP, ABTS, and RB5) [33]. Liers et al. [7] observed that all DyPs tested showed rather acidic pH optima (pH 1.4–2.5) for oxidizing non-phenolic aromatics.

These differences in biochemical properties of DyP isoenzymes might be attributed to their divergent evolutionary origin. Furthermore, DyP isoenzymes had complementary effects on different pH values, the combination of *Il*DyP2 and *Il*DyP1 isoenzymes might result in wider pH range for efficient lignin degradation.

The catalytic properties of *Il*DyP2 and *Il*DyP1 for classic fungal heme peroxidases substrates ABTS, DMP, and guaiacol were presented in Table 3. *Il*DyP2 and *Il*DyP1 exhibited significant differences in catalytic efficiencies for different substrates. *K*_m_ of *Il*DyP1 toward three substrates was higher than that of *Il*DyP2, suggesting that *Il*DyP2 had higher affinity for three substrates. *Il*DyP2 and *Il*DyP1 had similar activity for ABTS and guaiacol, while *Il*DyP2 had higher activity for DMP than for *Il*DyP1. *Il*DyP2 had sixfold and threefold higher catalytic efficiencies than *Il*DyP1 for ABTS and guaiacol. This suggested that *Il*DyP2 had better substrate fitting at the oxidation site, which was in accordance with the lack of activity of *Il*DyP1 on the recalcitrant dye RB5 [10]. In comparison with other typical fungal heme peroxidases, *Il*DyP2 and *Il*DyP1 displayed higher catalytic efficiencies for the oxidation of ABTS and DMP [34, 35].

**Table 3:** Kinetic constants for oxidation of ABTS, DMP, and guaiacol by *Il*DyP2 and *Il*DyP1 from *I. lacteus*

Substrate	pH	*Il*DyP	*K*_m_ [μM]	*k*_cat_ [s^−1^]	*k*_cat_/*K*_m_ [s^−1^ M^−1^]
ABTS	3	II	125.0 ± 1.4	396.2 ± 0.0	(3.2 ± 0.0) × 10^6^
	4	I	693.1 ± 8.5	356.3 ± 11.2	(5.1 ± 0.1) × 10^5^
DMP	3	II	1631.7 ± 10.9	560.8 ± 19.3	(3.4 ± 0.2) × 10^5^
	4	I	892.7 ± 81.5	198.8 ± 12.6	(2.2 ± 0.1) × 10^5^
Guaiacol	3	II	229.1 ± 15.9	49.7 ± 0.9	(2.2 ± 0.1) × 10^5^
	4	I	592.1 ± 53.0	49.2 ± 1.5	(8.3 ± 0.5) × 10^4^

**Fig. 6:**
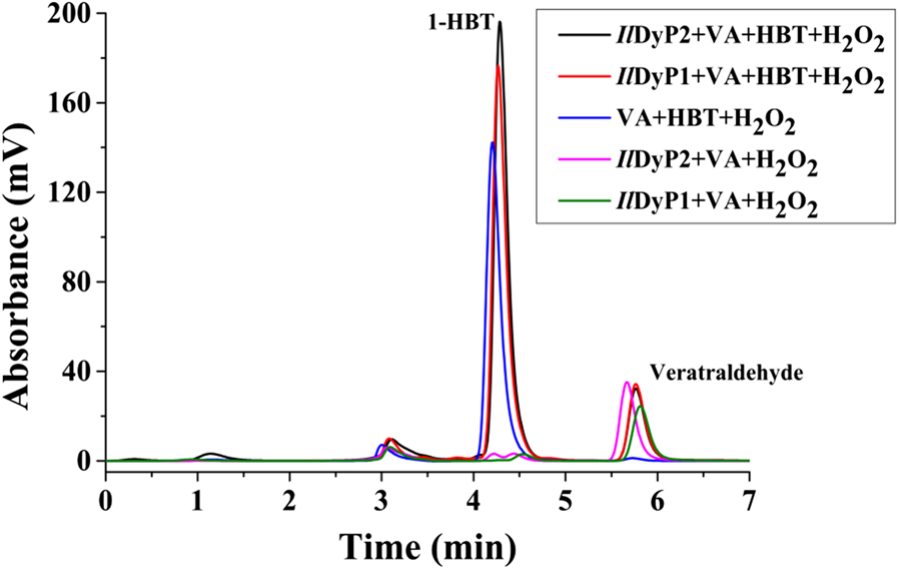
Oxidation of the non-phenolic lignin model compound, veratryl alcohol, in the absence or the presence of the mediator 1-HBT by *Il*DyP2 and *Il*DyP1 at 30 °C for 24 h

In addition, the data shown in Fig. 5 revealed that the substrate specificities of *Il*DyP2 and *Il*DyP1 were similar to that of classic fungal heme peroxidases. *Il*DyP2 and *Il*DyP1 were both able to decolorize the high redox potential anthraquinone dye RB19, which is the common feature of all DyP-type peroxidases [8]. Notably, *Il*DyP2 had the ability to decolorize the recalcitrant dye RB5, while *Il*DyP1 could not decolorize RB5. Although *Il*DyP2 and *Il*DyP1 could not oxidize Mn^2+^ to Mn^3+^, they could catalyze the oxidation of typical peroxidases substrates ABTS as well as phenolic lignin compounds DMP and guaiacol. Moreover, they exhibited low activity to oxidize non-phenolic lignin compound VA through peroxidase activity, belonging to the classic high redox potential peroxidases LiP and VP [2, 36]. It was also reported that other fungal DyPs can oxidize VA with very low activities [7, 35]. DyP in *I. lacteus* CCBAS 238 showed low activities for VA and RB5 [16]. In this study, we observed that mediator 1-HBT could promote VA degradation by *Il*DyP1. This phenomenon is similar to the oxidation of a non-phenolic lignin model compound by the laccase/1-HBT redox system [6, 37]. With 1-HBT as mediator, the oxidation rate of VA to veratraldehyde increased by 29.4% (Fig. 6). Considering their catalytic versatility, *Il*DyP2 and *Il*DyP1 might be a part of the alternative biocatalytic system for lignin degradation by *I. lacteus*, combining the catalytic properties of heme peroxidases and laccase.

Some proteins in DyP family have low amino acid sequence identity (lower than 15%), and significant differences in catalytic efficiency (*k*_cat_/*K*_m_) with a few orders of magnitude [38]. Bacterial DyPs possess a lower oxidizing ability than fungal DyPs, oxidizing only less recalcitrant phenolic lignin model compounds and monophenolic substrates [39]. However, Chen et al. found DyP from *Thermomonospora curvata* showed high catalytic efficiency with ABTS, close to that of fungal DyPs [40]. Except for dye decolorization, DyP from *Raoultella ornithinolytica* OKOH-1 can directly decolorize melanin, and immobilization can improve its activity and stability [41]. Whereas only a limited number of DyPs were purified and characterized. Further studies are still needed to assess the precise physiological roles and catalytic properties of DyPs, including fungal DyPs.

## Conclusions

*Irpex lacteus* CD2 grown in lignin medium secreted DyPs as the main extracellular ligninolytic enzymes. Transcriptomics analysis revealed that DyPs- and H_2_O_2_-generating enzymes including AOX, POX, and GLOX were coordinately expressed for efficient lignin degradation. Moreover, *Il*DyP2 and *Il*DyP1 could catalyze the oxidation of typical peroxidases substrates ABTS, phenolic lignin compounds DMP, and guaiacol as well as non-phenolic lignin compound VA. *Il*DyP1 could enhance the oxidation of non-phenolic lignin compound VA in the presence of mediator 1-HBT, the same as with Lac. These results proved for the first time that DyPs might depolymerize lignin when lacking classic heme peroxidases such as MnP, LiP, and Lac. DyPs could display different catalytic features of different peroxidases to different substrates, combining the catalytic properties of classic heme peroxidases and Lac. Therefore, DyPs may form an important constituent of the ligninolytic system in wood-decaying fungi.

## Materials and methods

### Chemicals

Alkali lignin, 2,2′-azino-bis (3-ethylbenzothiazoline-6-sulfonic acid) (ABTS), 2,6-dimethylphenol (DMP), guaiacol, reactive black 5 (RB5), veratryl alcohol (VA), and 1-hydroxybenzotriazole (1-HBT) were purchased from Sigma-Aldrich (St. Louis, MO). Reactive blue 19 (RB19) was purchased from Sinopharm Chemical Reagent Company (Beijing, China).

### Strain and culture conditions

*Irpex lacteus* CD2 [14] was maintained at 4 °C on potato dextrose agar plate. The inoculum was precultured in potato dextrose broth for 7 days at 28 °C, then 10% (v/v) inoculum was transferred into the modified Kirk’s medium, and shaken at 150 rpm. The Kirk’s medium contained: alkali lignin (or glucose) as the sole carbon source, 10 g/L; ammonium tartrate, 0.2 g/L; KH_2_PO_4_, 2 g/L; MgSO_4_·7H_2_O, 0.71 g/L; CaCl_2_, 0.1 g/L; and 70 mL trace element solution. The trace element solution contains NaCl, 1 g/L; CoCl_2_·6H_2_O, 0.184 g/L; FeSO_4_·7H_2_O, 0.1 g/L; ZnSO_4_·7H_2_O, 0.1 g/L; CuSO_4_, 0.1 g/L; H_3_BO_3_, 0.01 g/L; Na_2_MoO_4_·2H_2_O, 0.01 g/L; KAl(SO_4_)_2_·12H_2_O, 0.01 g/L; and nitrilotriacetic acid, 1.5 g/L.

### Enzymatic assays

Total ligninolytic enzyme activities were measured by monitoring the oxidation of ABTS (*ε*_420_ = 36,000 M^−1^ cm^−1^) at 420 nm, in 50 mM sodium tartrate buffer (pH 4.0) containing 1 mM ABTS, 1 mM Mn^2+^, and 0.1 mM H_2_O_2_. Manganese-independent peroxidase activity was also determined by ABTS oxidation in the absence of Mn^2+^. DyP activity was assayed by the decolorization of an anthraquinone dye RB19 (*ε*_595_ = 10,000 M^−1^ cm^−1^) at 595 nm. The reaction was performed in the same buffer containing 125 μM RB19 and 0.1 mM H_2_O_2_. One unit of enzyme activity was defined as the amount of enzyme that oxidized 1 μmol of ABTS or RB19 per minute at 25 °C.

### Secretome analysis

The extracellular enzymes of *I. lacteus* CD2 grown in alkali lignin at different periods of time were collected and concentrated by 80% ammonium sulfate [14]. The concentrated proteins were separated by one-dimensional SDS-PAGE, and the main bands on the third day were excised from the gel, digested with trypsin, and identified by nano LC–MS/MS. The peptides were separated in a reverse-phase C18 column, 0.18 mm × 100 mm, 5 μm particle size (Thermo). The mobile phases were A (water) and B (acetonitrile) containing 0.1% formic acid [22]. The flow rate was maintained at 300 nL/min. The phase B gradient was started at 3%, followed by a linear gradient to 8% in 1 min, 8–40% in 5 min, 40–85% in 1 min, and held there for 1 min. All MS/MS spectra were searched using PEAKS software against *I. lacteus* CD2 protein database using the following criteria [42]: enzyme trypsin; fixed modification of cysteine (+ 57.02 Da); and variable modification of methionine (+ 15.99 Da).

### Transcriptome analysis

*Irpex lacteus* CD2 was grown in the modified Kirk’s medium containing lignin or glucose as carbon source. The total RNA was extracted from mycelia on days 3 and 6 using the TRIZOL reagent (Invitrogen, Waltham, MA) according to the manufacturer’s instructions. The total RNA was sent to Annoroad Genomics (Beijing, China) for sample preparation and sequencing. All samples were in duplicate. The cDNA was synthesized and prepared for sequencing using the Illumina mRNA-Seq Sample Prep Kit (San Diego, CA). The samples were run on independent lanes, and paired-end sequences of 150 bp were obtained at 4 Gb clean data for each sample using the Illumina Hiseq 2500. The raw reads were trimmed and filtered using Trimmomatic software to remove adapters and low-quality bases [43]. Then clean reads were assembled into transcripts using TopHat and Cufflinks with the *I. lacteus* CD2 genome as a Ref. [14, 44]. All sequences of transcripts were extracted from reference sequence using gffread from cufflinks pipeline. The gene expression levels were conducted using the fragments per kilobase of exon per million fragments (FPKM) mapped method [45], and read counts were analyzed for differential expression using DESeq with a *q* value < 0.05 [46].

### Purification and characterization of *Il*DyPs

The liquid cultures of *I. lacteus* CD2 grown in alkali lignin for 3 days were collected and concentrated by 80% ammonium sulfate at 4 °C. 20 mM sodium acetate buffer (pH 5.0) was used to dissolve the pellets and dialyzed using 30-kDa cutoff membrane. Then *Il*DyPs were purified using a HiTrap Q HP anion exchange column (GE Health, Fairfield, CT) pre-equilibrated with the same acetate buffer. The *Il*DyPs were eluted with a linear gradient of 0–1.0 M NaCl, and fractions containing active enzymes were pooled after SDS-PAGE. Meanwhile, the bands were excised and identified by peptide mass fingerprinting.

To determine the optimal pH, 50 mM sodium tartrate buffers with pH ranging from 2.0 to 7.0 were used for all substrates including ABTS, DMP, guaiacol, VA, RB19, and RB5 at 25 °C. The maximum activities of *Il*DyP2 and *Il*DyP1 were considered to be 100%. For catalysis properties, the reactions were performed in optimal pH at 25 °C using 50–4000 μM substrates by monitoring corresponding oxidation products. The nonlinear least square fitting method was used to calculate the *K*_m_, *k*_cat_, and *k*_cat_/*K*_m_ parameters of *Il*DyP2 and *Il*DyP1 using the GraphPad Prism 5 software.

1-HBT was used as the mediator in evaluating the abilities of *Il*DyP1 and *Il*DyP2 to oxidize the non-phenolic lignin compound VA. The oxidation of VA was performed in 50 mM sodium tartrate buffer (pH 4.0 or pH 3.0) containing 1 mM VA, 1 mM 1-HBT, 0.1 mM H_2_O_2_, and 0.5 U/mL *Il*DyP1 or *Il*DyP2, without 1-HBT or DyP as corresponding control. The reaction proceeded at 30 °C. After 24 h, the reaction products were analyzed by HPLC using a reverse-phase C18 column, 4.6 mm × 250 mm, 5-m particle size (Waters XTerra). The isocratic elution condition was performed with 55% methanol containing 0.1% formic acid at a flow rate of 1 mL/min. The elution peaks were monitored at 310 nm.

## Additional files

**Additional file 1.** Proteins identification from *I. lacteus* secretome in kirk’s medium supplemented with lignin on the third day.

**Additional file 2.** Transcript levels of *I. lacteus* genes encoding carbohydrate active enzymes in GLU3d, GLU6d, LIG3d and LIG6d.

**Additional file 3.** Transcript levels of *I. lacteus* genes involved in mobilizing and recycling nitrogen in GLU3d, GLU6d, LIG3d and LIG6d.
